# Littoral Cell Angioma of the Spleen: A Case Report

**DOI:** 10.7759/cureus.37137

**Published:** 2023-04-04

**Authors:** Raghunath Ramanarasimhaiah, Robert Colef, Nfn Kiran, Kokila Mody

**Affiliations:** 1 Pathology and Laboratory Medicine, Northwell Health, Staten Island, USA; 2 Pathology and Laboratory Medicine, Northwell Health, New York, USA

**Keywords:** hamartoma, hemangioma, littoral cell angioma, vascular tumors, primary neoplasia of the spleen

## Abstract

Littoral cell angioma (LCA) is a rare, primary vascular tumor of the spleen that originates from the cells lining the venous sinuses of the spleen. Around 150 cases have been reported worldwide, with most reported cases of LCA being non-malignant but with unspecified malignant potential. As of 2022, three cases of malignant LCA have been reported. A 75-year-old male with a history of monoclonal gammopathy of uncertain significance presented with left upper outer quadrant abdominal pain. Ultrasound (US) scan showed a 10.5 cm round, circumscribed mass lesion, with hyperechoic foci, occupying the posterolateral aspect of the spleen. US-guided core needle biopsy of the mass revealed a diagnosis of “atypical cells present, suggestive of vascular neoplasm of the spleen,” which was based on histologic and immunohistochemistry characteristics. Due to the size of the lesion, a malignant neoplasm was suspected, and a splenectomy was performed. Histological and immunohistochemical features of the splenic lesion returned a final diagnosis of benign LCA.

## Introduction

Littoral cell angioma (LCA) was first described and named so by Falk in 1991 [1]. It is a rare, primary vascular tumor of the spleen arising from cells lining the splenic red pulp venous sinuses that show features of combined endothelial and histiocytic differentiation. Around 150 cases have been reported worldwide with most reported cases being benign but with unspecified malignant potential [1-6]. LCA is more prevalent in Asia and South America [5-8]. It usually presents as isolated splenomegaly with a single lesion or multiple recurrent lesions. Although the etiology of LCA remains mostly unknown, an association between LCA and autoimmune or metabolic disorders has been reported [5-11]. There are no specific imaging findings of LCA, and the differential diagnosis after imaging includes hamartoma, lymphoma, Kaposi’s sarcoma, lymphangioma, and hemangioma. The final diagnosis of LCA is based on pathologic findings of anastomosing vascular channels lined by tall, cuboid, plump endothelial cells, some of which demonstrate hemosiderin pigments; of note, nuclear atypia and mitotic activity are not present. Immunohistochemistry aids in the diagnosis of LCA and has the following characteristics: strong positivity for CD31; focal positivity for CD68, factor VIII, CD8, and lysozyme; and negative reaction for CD34. Additionally, some tumor cells have shown a positive reaction with periodic acid-Schiff staining [12].

## Case presentation

**Figure 1:**
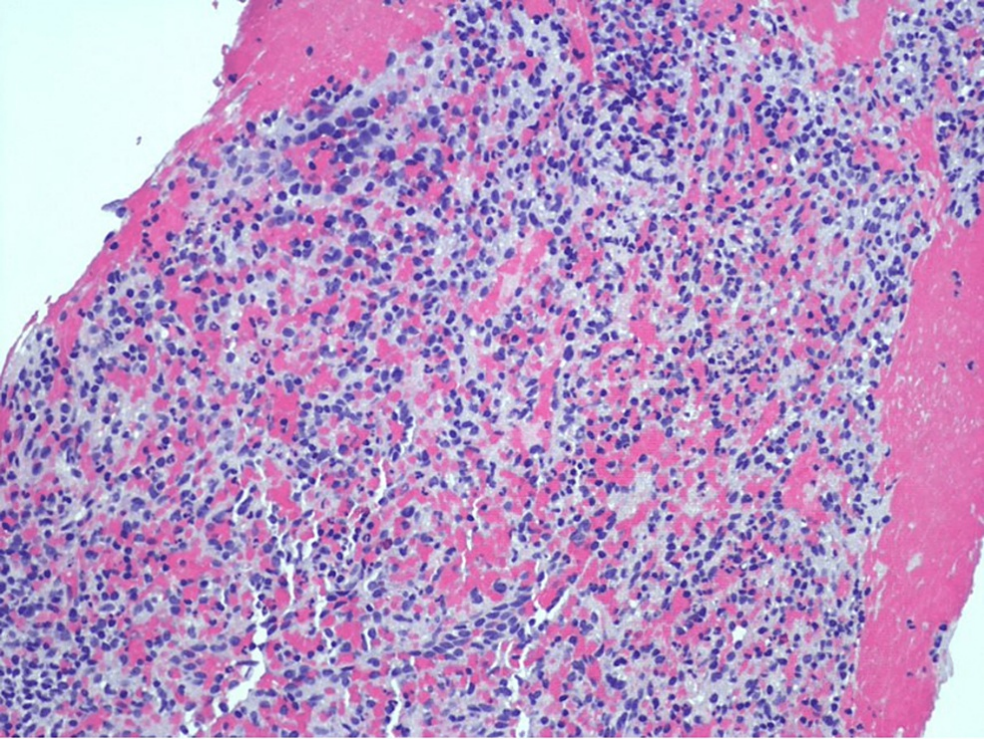
Fine needle aspiration biopsy of splenic mass (hematoxylin and eosin, 200×).

A 75-year-old white male with a history of monoclonal gammopathy of uncertain significance presented with vague abdominal pain. On ultrasound scan, a 10.5 cm, round, circumscribed mass lesion, with hyperechoic foci, occupying the posterolateral aspect of the spleen was identified. He underwent an ultrasound-guided core needle biopsy, which histologically showed lymphoid tissue, intermixed with a subpopulation of spindle to oval-shaped atypical cells (Figure 1).

**Figure 2:**
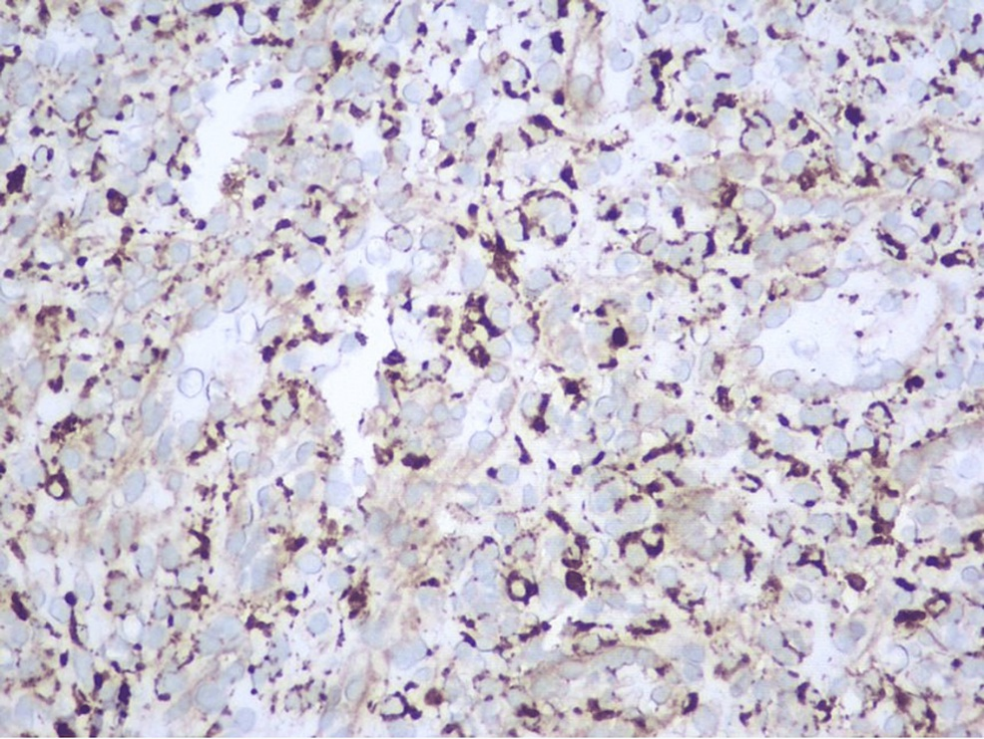
CD68 stain (400×).

Immunohistochemical stains were positive for CD31, CD4, CD8 (focally), CD34 (focally), and CD68 (Figure 2).

**Figure 3:**
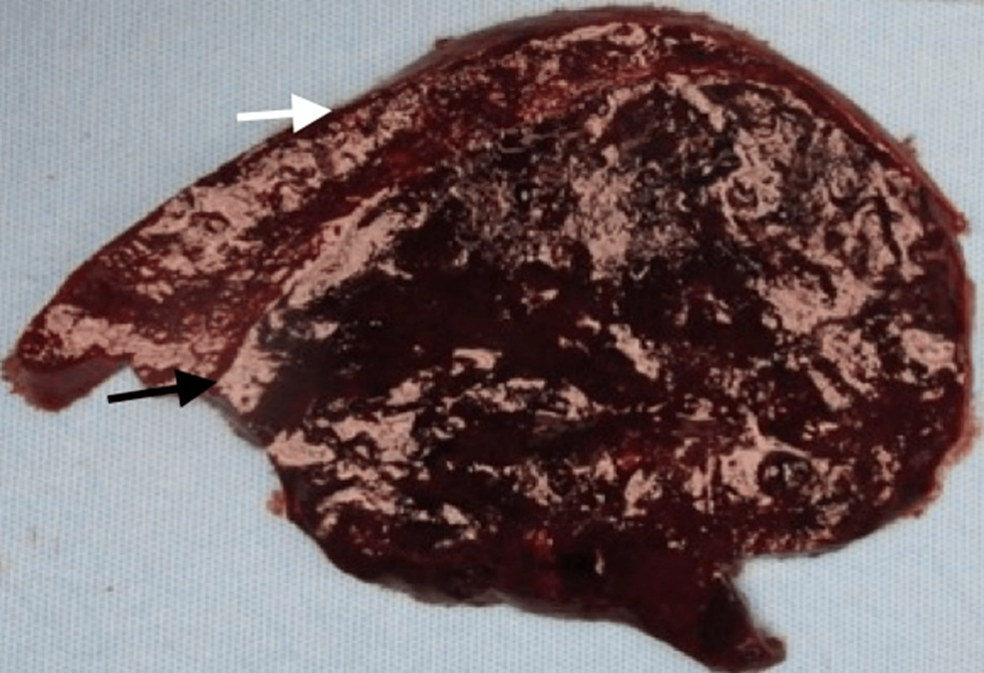
Gross image, cross-section of the spleen. The black arrow highlights the lesion is well-circumscribed and dark red, and the white arrow highlights the cut surface of normal splenic parenchyma.

Flow cytometric analysis did not show any evidence of lymphoma. Based on the preceding findings, the possibility of a vascular neoplasm of the spleen was reported with a differential diagnosis of LCA and hamartoma of the spleen. Due to the size of the lesion and the biopsy diagnosis, a splenectomy was performed. The preoperative MRI of the spleen confirmed the presence and size of the splenic lesion. Grossly, the spleen weighed 826 g (normal 28-226 g) [13] and measured 11 × 10.5 × 4.5 cm with a well-circumscribed, dark-red, soft mass in the posterolateral aspect of the spleen (Figure 3). The remaining cut surfaces showed unremarkable red splenic parenchyma.

**Figure 4:**
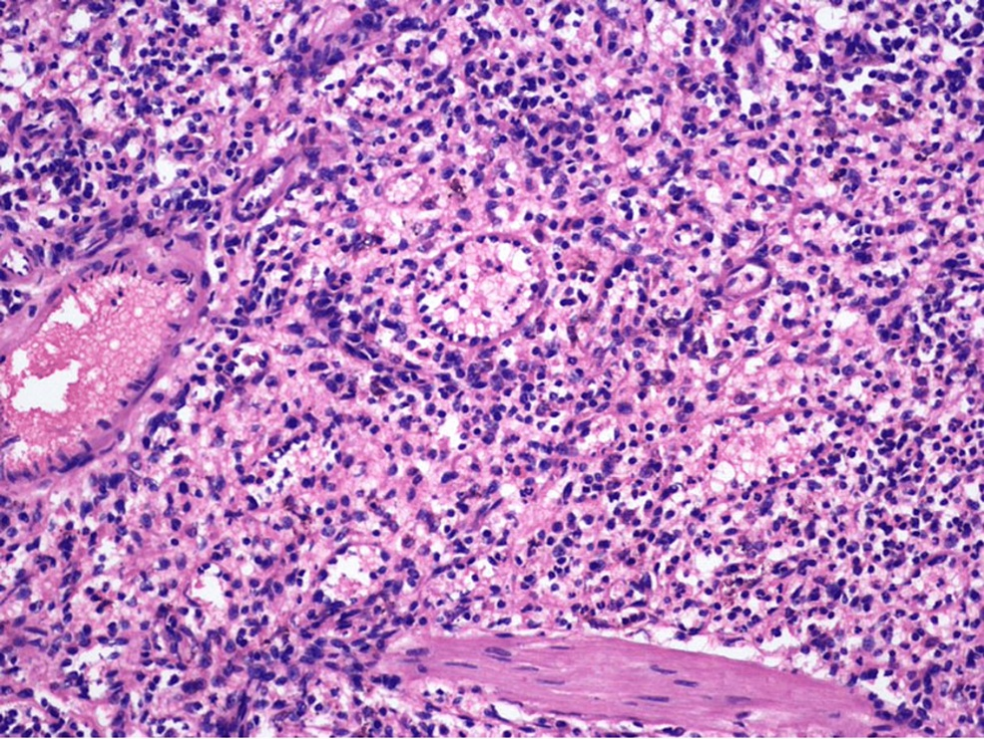
Microscopy of splenectomy specimen (hematoxylin and eosin, 200×).

**Figure 5:**
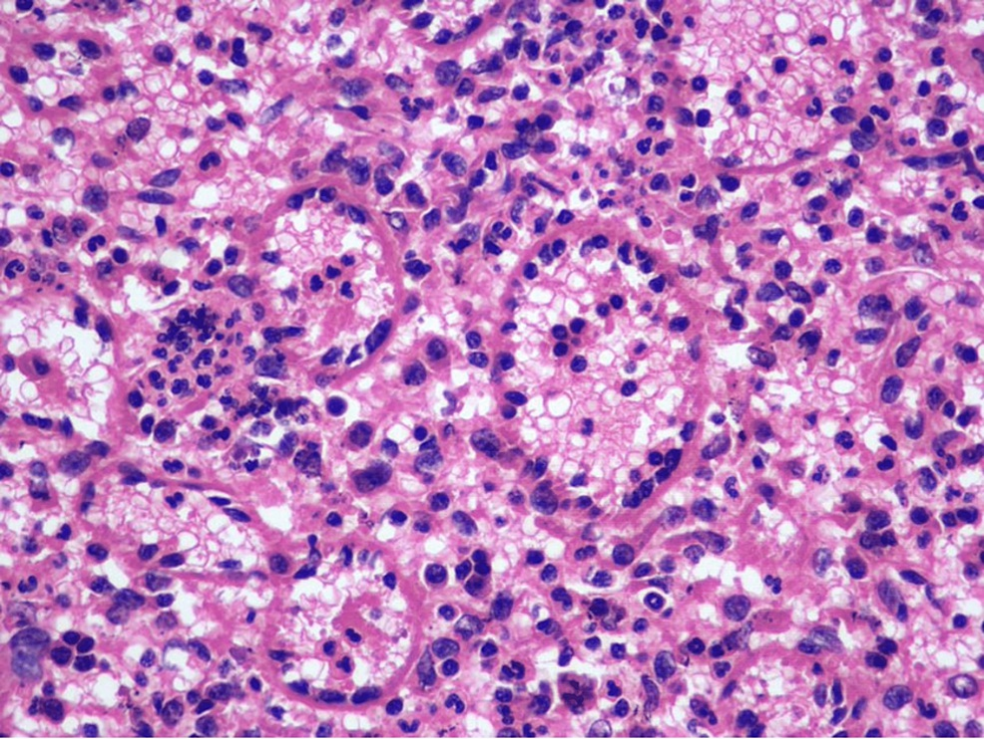
Microscopy of splenectomy specimen (hematoxylin and eosin, 400×).

On microscopy, anastomosing, tortuous vascular channels with irregular lumina containing exfoliated cells and lined by plump cells with oval nuclei were seen (Figures 4, 5).

**Figure 6:**
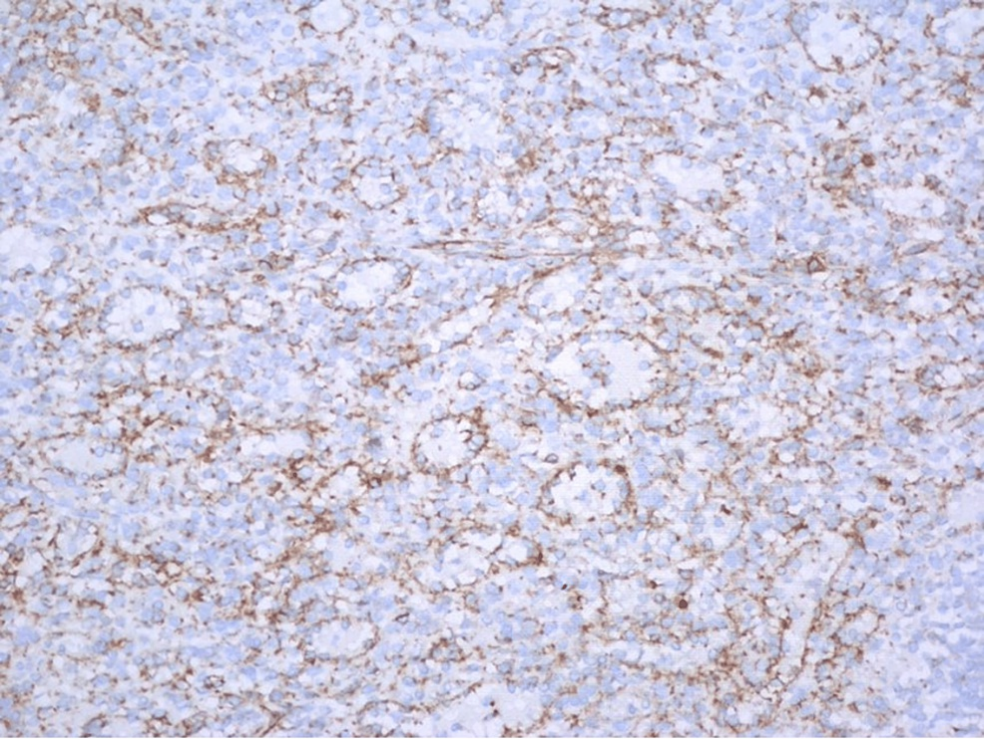
CD31 stain (200×).

Immunohistochemical stains were positive for CD68 and CD31 (Figure 6).

Immunohistochemical stains for CD34, CD4, CD8, CD21, and CD117 were negative. Based on these findings, a final diagnosis of LCA was made.

## Discussion

LCA is a rare, primary neoplasm of the spleen with unspecified malignant potential, affecting both men and women equally. Often, LCA is an incidental imaging finding but can present with vague abdominal pain or splenic rupture. The morphologic and immunophenotypic features of LCA that reflect the dual endothelial and histiocytic potential of splenic sinus-lining cells are different from other angiomas showing co-expression of both macrophage markers CD68 and lysozyme, as well as endothelial markers FVIII and CD31 [12]. Around 150 cases have been reported worldwide. An association between LCA and autoimmune or metabolic disorders has been reported [5-11]. Additionally, LCA has been shown to recur after surgical excision and leads to distant metastasis [13-16]. Imaging studies such as MRI and CT are inadequate in diagnosing LCA due to the difficulty of differentiating it from other splenic neoplasms [17]. Although LCA is considered a benign entity, littoral cell hemangioendothelioma and littoral cell angiosarcoma are considered malignant subtypes of LCA. Littoral cell hemangioendothelioma can show nuclear atypia, necrosis, and a high proliferation index (Ki-67+). The presence of solid clear cell areas differentiates it from LCAs [18]. Rosso et al. described littoral cell angiosarcoma as showing solid tumor nests, cytological atypia, mitotic activity, and lack of CD68 staining compared to LCA [19]. In addition, the proliferation index is higher in angiosarcoma [20]. Given that LCA does have malignant subtypes and that imaging provides little help in its diagnosis, surgical excision becomes necessary.

## Conclusions

LCA is a rare, primary neoplasm of the spleen with unspecified malignant potential. Because LCA has malignant subtypes with imaging providing little help in its diagnosis, surgical excision becomes necessary. Patients diagnosed with LCA may require closer follow-up for possible recurrences and tumor dissemination. Taking the above facts into consideration, reporting this neoplasm will be beneficial to its management.
